# Lymph Node Involvement and the Clinical Stage as Predictors of the Survival of Patients With Adenoid Cystic Carcinoma of the Head and Neck: A Systematic Review and Meta-Analysis

**DOI:** 10.7759/cureus.30780

**Published:** 2022-10-27

**Authors:** Mazin Alsarraj, Sami M Alshehri, Abdulrahman Qattan, Abdelelah Mofti, Lana Wazqer, Sumayiah Bukhari, Aisha Shamsaldin, Rahaf Rajab

**Affiliations:** 1 Otolaryngology - Head and Neck Surgery, King Fahad General Hospital, Jeddah, SAU; 2 Otolaryngology - Head and Neck Surgery, King Abdullah Medical Complex, Jeddah, SAU; 3 Otolaryngology - Head and Neck Surgery, University of Jeddah, Jeddah, SAU

**Keywords:** stage, salivary gland tumor, head and neck tumors, lymph node status, adenoid cystic carcinoma (acc)

## Abstract

In the treatment of various patients, the presence of lymphovascular invasion is a prognostic determinant, often taken into account by surgeons and oncologists. The exact frequency and prognostic impacts of this microscopic event in adenoid cystic carcinoma (ACC) patients are, however, not clear. This systematic review and meta-analysis aimed to investigate the lymph node involvement and the clinical stage of cancer as predictors of ACC prognosis. A systematic search was conducted covering a number of databases, including PubMed, Science Direct, Google Scholar, Web of Science, and EBSCO. A total of three studies were included in this analysis, with 591 participants, 247 of whom were males. Lymph node involvement and clinical stage were demonstrated as significant bad prognosis factors among ACC patients (HR = 1.48, 95% CI, 1.00, 1.96; P<0.0001). We found that lymph node involvement and clinical stage of the cancer are both significant predictors of bad prognosis of ACC.

## Introduction and background

Adenoid cystic carcinoma (ACC) is a rare malignant tumor that affects major and minor salivary glands, lacrimal glands, ceruminous glands, and occasionally cervix, breast, and lung [1]. It accounts for fewer than 1% of all head and neck malignancies and 10% of all salivary gland neoplasms [2,3]. ACC occurs more often in the minor salivary glands than in the major salivary glands that are dispersed throughout the submucosa of the nasal cavity, paranasal sinuses, and oral pharynx, larynx, trachea, lungs, and middle ear cavity [2-5]. The paranasal sinus is the most common site of minor salivary gland involvement [1,6,7]. ACC is responsible for 5% to 15% of all malignant tumors of the nasal cavity and paranasal sinuses [8].

ACC of the nasal cavity and paranasal sinuses is a slowly progressive and relentless tumor that tends to recur locally and metastasize distantly [9,10]. Recurrence and metastasis can occur even decades after treatment of the primary tumor [11]. The steadfastness of this tumor is partially attributable to its ability to extend through submucosal and fibrous tissue planes around the primary site and its tendency for perineural spread along major and minor nerves [3,12]. Therefore, treatment of ACC of the nasal cavity and paranasal sinuses is highly challenging, with current therapy limited to surgery and/or radiation, and no reliable chemotherapeutic options available for long-term disease control [13,14].

Because of the slow and persistent clinical course of ACC of the nasal cavity and paranasal sinuses, on initial evaluation, most patients have advanced disease, which makes the treatment more difficult [15]. Therefore, it is essential to identify the prevalence of disease to detect the patient during the early stage and start treatment early. According to many studies, ACC of the nasal cavity and paranasal sinuses is more prevalent in middle-aged adults; however, a demographic analysis presented that most patients were diagnosed in the sixth decade or later [16-18]. Most previous studies conducted on ACC of nasal cavity and paranasal sinuses identified determinants of outcomes for ACC, including stage, cervical lymph node metastases, margin status, perineural invasion, and minor salivary gland site of origin, although further investigations are needed to identify the prevalence of ACC in the nasal cavity and paranasal sinuses [19,20-23]. Our study aims to estimate the risk of developing ACC based on lymph node involvement and the clinical stage of the tumor.

## Review

Materials and methods

Study Design and Duration

This is a systematic review and meta-analysis that was carried out during October 1-20, 2021. Our study focused on examining lymph node involvement and clinical stage as risk factors for ACC in the published literature.

Search Strategy

A systematic search was conducted covering a number of databases, including PubMed, Science Direct, Google Scholar, Web of Science, and EBSCO. The search was confined to articles published in the English language and tailored for each database. The following terms have been used in our search: “ACC”, “Adenoid cystic carcinoma”, “Lymph node involvement”, “Lymph node metastasis”, “LN metastasis”, “clinical stage”, “Cancer staging”, “risk factor”, and “liability”, “survival rate”. Only full texts, accessible articles, human trials, and English articles were chosen. Table 1 shows the selection criteria.

**Table 1:** Selection criteria

Inclusion criteria	Exclusion criteria
Prospective studies	Studies conducted in languages other than English
Studies reporting results covering the impact of lymph node involvement and/or clinical stage of adenoid cystic carcinoma	Inaccessible studies
	Retrospective studies
In vivo studies	Case reports and case series

Data Extraction

Rayyan (Qatar Computing Research Institute, Doha) was used throughout the selection process to identify duplicates and remove them and, in addition, to choose our included articles following the inclusion and the exclusion criteria [24]. We started with assessing titles for compatibility, followed by examining abstracts, and finally, full-text review. A data extraction sheet was created to pool the data from the included articles. The sheet included titles, authors, study year, study design, population type, participants’ number, age range and mean, number and percentage of male participants, cancer stage, and lymph node involvement. We overcame any disagreement through debate and discussion.

Risk of Bias Assessment

The Newcastle-Ottawa Scale (NOS) was used to evaluate the quality of the included studies through quantitative data synthesis [25].

Strategy for Data Synthesis

Data collected through the Excel sheet (Microsoft, Redmond) was used to generate the qualitative overview of the included studies. A qualitative synthesis of the pooled data was carried out regardless of the viability of the present meta-analyses. Only studies that included numerical results about the involvement of lymph nodes and the clinical stage of ACC were excluded.

Qualitative data synthesis for the condition of interest was performed using Review Manager software (Cochrane Collaboration, UK). The lymph node metastasis and clinical stage as risk factors for ACC were evaluated using random-effect meta-analysis. Heterogeneity was assessed using the I-square test, and publication bias was estimated through funnel plot and funnel plot symmetry.

Results

Search Results

**Figure 1:**
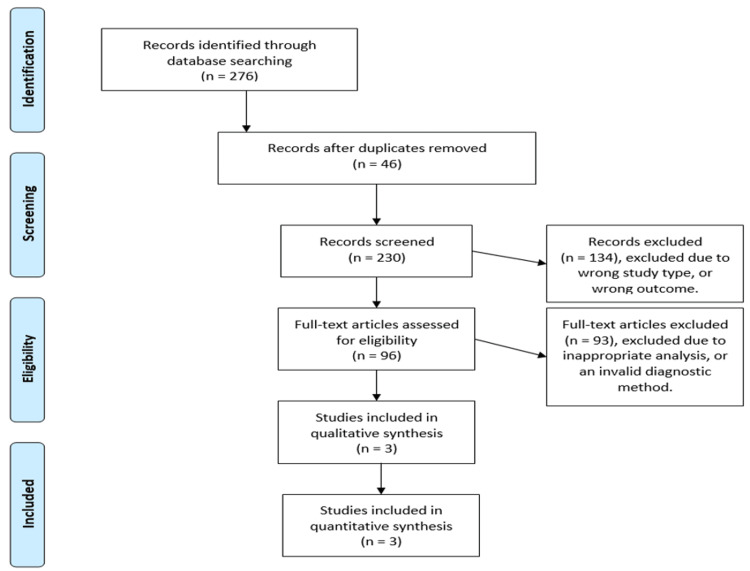
Preferred Reporting Items for Systematic Reviews and Meta-Analyses (PRISMA) flow chart presenting a summary of the selection process

A total of 276 articles have been retrieved using our systematic search, of which 46 duplicates were removed using Rayyan, and 230 studies were enrolled for title screening; of those 134 were excluded. The remaining 96 studies underwent abstract and full-text screening leaving only three studies to be included. Reasons for exclusion were irrelevant analyses, wrong outcome, wrong population, or unavailable data on the risk of developing ACC in the case of nodal involvement and depending on the clinical stage of cancer. Figure 1 illustrates the selection process.

Characteristics of the Included Studies

This systematic review and meta-analysis included three studies with 591 participants, 247 of whom were males. Their age ranged between 18 and 86 years. The three studies were prospective studies: one from France, another from Switzerland, and the third from Korea. The observation time ranged from 30 to 160 months. Table 2 describes the characteristics of included studies.

**Table 2:** Summary of the characteristics of the included studies

Study	Study design	Country	Total participants	Mean age	Males (%)	Condition	Treatment/observation time (months)
A prospective multicentre REFCOR study of 470 cases of head and neck Adenoid cystic carcinoma: epidemiology and prognostic factors [26]	Prospective study	France	470	54	41%	Adenoid cystic carcinoma	39
Clinical outcomes of head and neck adenoid cystic carcinoma patients treated with pencil beam-scanning proton therapy [27]	Prospective study	Switzerland	35	45.4	54.3%	Adenoid cystic carcinoma	30
Long-term treatment outcomes and prognostic features in adenoid cystic carcinoma of the head and neck [28]	Prospective study	Korea	86	50 (median)	43%	Adenoid cystic carcinoma	160.44 months (median)

Lymph Node Involvement and Clinical Stage as Risk Factors for ACC

**Figure 2:**
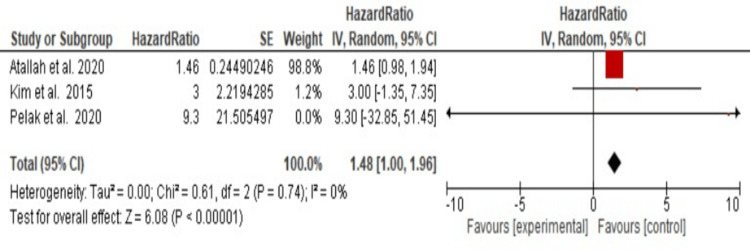
Forest plot of the effect of lymph node involvement and clinical stage of cancer on the development of adenoid cystic carcinoma The red box represents the quantitative value of each entry. The black diamond symbol represents the pooled effect estimate with confidence intervals. Studies included [26,28,29].

Three studies investigated the incidence of ACC in the presence of nodal involvement and the association with the clinical stage. Lymph node involvement and clinical stage have been demonstrated as significant factors increasing the risk of death among ACC patients (HR = 1.48, 95% CI, 1.00, 1.96; P<0.0001). Significant heterogeneity between the studies was not detected (I^2^=0%, P=0.74; Figure 2).

Publication Bias

**Figure 3:**
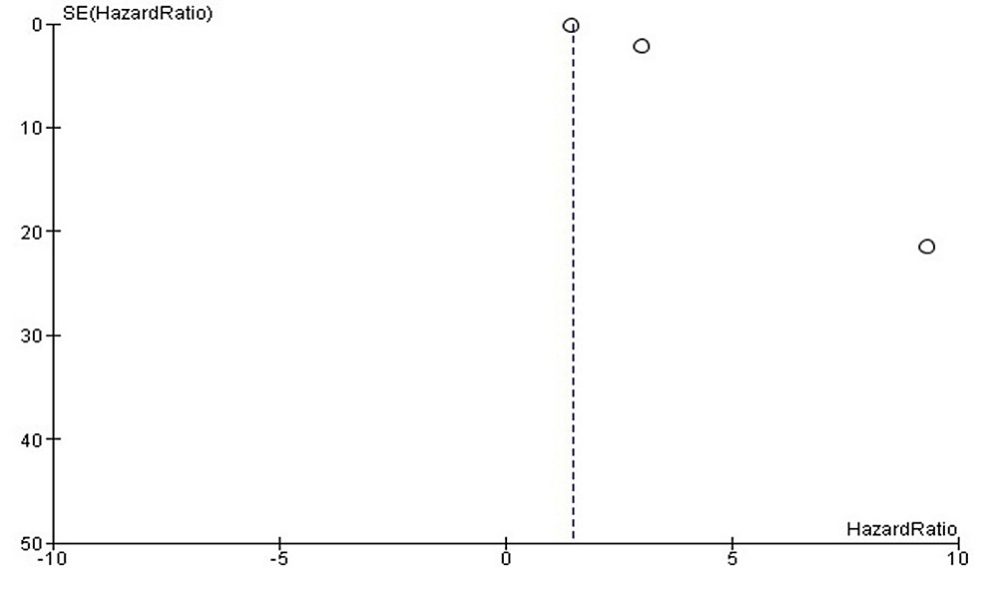
Funnel plot of publication bias detection Circles represent plotted data from each entry on the funnel plot.

Visual observation of the funnel plot reveals an asymmetrical distribution of lymph node involvement and clinical stage as potential risk factors for ACC (Figure 3).

Discussion

In this systematic review and meta-analysis, we investigated lymph node involvement and the clinical stage of cancer as prognostic factors for ACC. The results have shown, however, that both are predictors of bad prognosis in the case of ACC. The strength of the study stems from its uniqueness and meticulously designed systematic search. Our results, however, should be interpreted carefully because of the lack of available data concerning the topic and the few numbers of studies involved.

ACC has been extensively studied in previous studies that supported the claim that lymph node involvement and the clinical stage of cancer have bad prognostic effects on ACC [29,30]. A number of studies have shown that a microscopic finding is associated with poorer clinical behaviors, such as prostate, bladder, lung, and kidney cancers, as well as other malignant salivary glands diseases, such as myoepithelial carcinoma and ex pleomorphic adenoma carcinoma [31-34].

Tang et al. found an independent prognostic determinant for the lymphovascular invasion in ACC [35]. Oplatek et al. showed a significant association with recurrence [21]. While these results point to a more aggressive condition associated with lymphovascular invasions, none of the available studies describe the lymphovascular invasion in ACC as its main objective, which is always the secondary finding.

Thus, few authors have studied the association between lymphovascular invasion and clinicopathological parameters while some did not check on the original slides, and none used ancillary techniques for the better identification of lymphovascular invasions, such as immune histochemistry. Moreover, because we do not know what types of vessels are invaded, either blood or lymphatic vessels, the profile of tumor emboli has not been well described. Nor have the vessel’s morphological characteristics been described. An original study is, therefore, necessary in order to assess the prognostic potential of lymphovascular invasion.

The small sample size used and the short follow-up time described for most of the studies are other constraints in the available studies. Moreover, multivariate statistical analysis has not always been conducted, and, consequently, it remains unclear whether lymphovascular invasion is a significant predictor once other significant variables such as tumor grade are controlled. The heterogeneity for the meta-analysis nevertheless showed that there were very low variables between studies, and the results were reliable.

## Conclusions

In addition to the prognostic significance of lymphovascular invasion and the clinical stage of cancer, several methodological limitations of the studies available were also demonstrated by the current systematic evaluation and meta-analysis, for patients affected by ACC of the head and neck. Therefore, we understand that future studies should determine how frequently and prognostically, by using immunomarkers towards the blood and lymphatic vessels, the infiltration of both types of the vessels would be of equal importance as well as the frequency of the lymphovascular invasion when only regular hematoxylin and eosin stains are misidentified.
